# Supplementary material for: Heparin-based hydrogel scaffolding alters the transcriptomic profile and increases the chemoresistance of MDA-MB-231 triple-negative breast cancer cells
Source: Biomater Sci. 2020 Feb 13;8(10):2786–96. doi: 10.1039/c9bm01481k (PMC7497406; doi:10.1039/c9bm01481k)
Supplement: Supplementary file 2 [file BM-008-C9BM01481K-s002.zip › Supplementary File 4/HP-B hydrogel + EGF (s)vControl/Pathways/my_analysis.Gsea.1545201170987/HALLMARK_XENOBIOTIC_METABOLISM.html]

Details for gene set HALLMARK\_XENOBIOTIC\_METABOLISM[GSEA]

|  || Dataset | expr.class.cls#HYDEGF\_versus\_CONTROL.class.cls#HYDEGF\_versus\_CONTROL\_repos |
| Phenotype | class.cls#HYDEGF\_versus\_CONTROL\_repos |
| Upregulated in class | CONTROL |
| GeneSet | HALLMARK\_XENOBIOTIC\_METABOLISM |
| Enrichment Score (ES) | -0.3097366 |
| Normalized Enrichment Score (NES) | -1.4269073 |
| Nominal p-value | 0.0 |
| FDR q-value | 0.032705594 |
| FWER p-Value | 0.363 |
Table: GSEA Results Summary

  

Fig 1: Enrichment plot: HALLMARK\_XENOBIOTIC\_METABOLISM      
 Profile of the Running ES Score & Positions of GeneSet Members on the Rank Ordered List

  

| PROBE | DESCRIPTION (from dataset) | GENE SYMBOL | GENE\_TITLE | RANK IN GENE LIST | RANK METRIC SCORE | RUNNING ES | CORE ENRICHMENT || 1 | ATOH8 | na |  |  | 9 | 3.823 | 0.0286 | No |
| 2 | LCAT | na |  |  | 61 | 3.169 | 0.0499 | No |
| 3 | CYP1A1 | na |  |  | 102 | 2.902 | 0.0699 | No |
| 4 | SLC12A4 | na |  |  | 282 | 2.410 | 0.0788 | No |
| 5 | SLC1A5 | na |  |  | 679 | 1.922 | 0.0726 | No |
| 6 | FBLN1 | na |  |  | 689 | 1.914 | 0.0867 | No |
| 7 | ASL | na |  |  | 812 | 1.812 | 0.0940 | No |
| 8 | UPP1 | na |  |  | 814 | 1.811 | 0.1077 | No |
| 9 | GCKR | na |  |  | 1188 | 1.579 | 0.1001 | No |
| 10 | CSAD | na |  |  | 1233 | 1.547 | 0.1096 | No |
| 11 | ID2 | na |  |  | 1377 | 1.479 | 0.1133 | No |
| 12 | GSS | na |  |  | 1693 | 1.361 | 0.1071 | No |
| 13 | LONP1 | na |  |  | 1846 | 1.312 | 0.1091 | No |
| 14 | HES6 | na |  |  | 2118 | 1.234 | 0.1042 | No |
| 15 | ABCC2 | na |  |  | 2277 | 1.188 | 0.1049 | No |
| 16 | PEMT | na |  |  | 2435 | 1.144 | 0.1054 | No |
| 17 | EPHA2 | na |  |  | 2860 | 1.038 | 0.0910 | No |
| 18 | AP4B1 | na |  |  | 3332 | 0.937 | 0.0734 | No |
| 19 | PINK1 | na |  |  | 3407 | 0.921 | 0.0765 | No |
| 20 | BCAR1 | na |  |  | 3435 | 0.916 | 0.0820 | No |
| 21 | PPARD | na |  |  | 3488 | 0.907 | 0.0862 | No |
| 22 | GAD1 | na |  |  | 3542 | 0.896 | 0.0902 | No |
| 23 | HACL1 | na |  |  | 3545 | 0.895 | 0.0969 | No |
| 24 | ACOX3 | na |  |  | 3685 | 0.869 | 0.0962 | No |
| 25 | CYP2E1 | na |  |  | 3918 | 0.827 | 0.0903 | No |
| 26 | ACP2 | na |  |  | 4914 | 0.666 | 0.0431 | No |
| 27 | FAH | na |  |  | 4997 | 0.653 | 0.0438 | No |
| 28 | POR | na |  |  | 5102 | 0.637 | 0.0432 | No |
| 29 | HGFAC | na |  |  | 5512 | 0.575 | 0.0261 | No |
| 30 | PYCR1 | na |  |  | 5858 | 0.524 | 0.0119 | No |
| 31 | AKR1C2 | na |  |  | 5924 | 0.513 | 0.0124 | No |
| 32 | ABCC3 | na |  |  | 6031 | 0.498 | 0.0106 | No |
| 33 | NMT1 | na |  |  | 6101 | 0.490 | 0.0107 | No |
| 34 | PLG | na |  |  | 6144 | 0.483 | 0.0122 | No |
| 35 | DHPS | na |  |  | 6243 | 0.470 | 0.0106 | No |
| 36 | XDH | na |  |  | 6841 | 0.391 | -0.0177 | No |
| 37 | NQO1 | na |  |  | 7079 | 0.362 | -0.0274 | No |
| 38 | AOX1 | na |  |  | 7085 | 0.361 | -0.0250 | No |
| 39 | CYFIP2 | na |  |  | 7182 | 0.347 | -0.0274 | No |
| 40 | CYP2S1 | na |  |  | 7315 | 0.328 | -0.0318 | No |
| 41 | MPP2 | na |  |  | 7404 | 0.316 | -0.0340 | No |
| 42 | SLC35D1 | na |  |  | 7417 | 0.314 | -0.0323 | No |
| 43 | ETS2 | na |  |  | 7527 | 0.301 | -0.0357 | No |
| 44 | LEAP2 | na |  |  | 7578 | 0.296 | -0.0361 | No |
| 45 | VTN | na |  |  | 7622 | 0.286 | -0.0362 | No |
| 46 | SERTAD1 | na |  |  | 7762 | 0.269 | -0.0414 | No |
| 47 | TTPA | na |  |  | 7868 | 0.257 | -0.0450 | No |
| 48 | MTHFD1 | na |  |  | 8285 | 0.210 | -0.0652 | No |
| 49 | SLC35B1 | na |  |  | 8447 | 0.196 | -0.0722 | No |
| 50 | GART | na |  |  | 8738 | 0.159 | -0.0862 | No |
| 51 | GCLC | na |  |  | 8925 | 0.131 | -0.0950 | No |
| 52 | CYP2J2 | na |  |  | 9266 | 0.094 | -0.1121 | No |
| 53 | ATP2A2 | na |  |  | 9352 | 0.081 | -0.1160 | No |
| 54 | CBR1 | na |  |  | 9495 | 0.076 | -0.1228 | No |
| 55 | FMO3 | na |  |  | 9524 | 0.072 | -0.1238 | No |
| 56 | GCNT2 | na |  |  | 9548 | 0.069 | -0.1245 | No |
| 57 | TPST1 | na |  |  | 9662 | 0.053 | -0.1300 | No |
| 58 | AHCY | na |  |  | 10060 | 0.011 | -0.1507 | No |
| 59 | DHRS7 | na |  |  | 10107 | 0.004 | -0.1531 | No |
| 60 | RAP1GAP | na |  |  | 10310 | -0.024 | -0.1635 | No |
| 61 | HMOX1 | na |  |  | 10412 | -0.037 | -0.1686 | No |
| 62 | KARS | na |  |  | 10741 | -0.071 | -0.1853 | No |
| 63 | PMM1 | na |  |  | 10773 | -0.076 | -0.1863 | No |
| 64 | SHMT2 | na |  |  | 10804 | -0.078 | -0.1873 | No |
| 65 | SLC6A6 | na |  |  | 10932 | -0.099 | -0.1932 | No |
| 66 | COMT | na |  |  | 11050 | -0.114 | -0.1985 | No |
| 67 | GNMT | na |  |  | 11056 | -0.115 | -0.1979 | No |
| 68 | SSR3 | na |  |  | 11435 | -0.151 | -0.2166 | No |
| 69 | PAPSS2 | na |  |  | 11849 | -0.211 | -0.2366 | No |
| 70 | FBP1 | na |  |  | 11864 | -0.213 | -0.2358 | No |
| 71 | APOE | na |  |  | 12183 | -0.241 | -0.2506 | No |
| 72 | BCAT1 | na |  |  | 12337 | -0.267 | -0.2566 | No |
| 73 | ENTPD5 | na |  |  | 12416 | -0.274 | -0.2586 | No |
| 74 | CNDP2 | na |  |  | 12494 | -0.286 | -0.2605 | No |
| 75 | NFS1 | na |  |  | 12509 | -0.288 | -0.2591 | No |
| 76 | MAN1A1 | na |  |  | 12647 | -0.305 | -0.2639 | No |
| 77 | RETSAT | na |  |  | 12706 | -0.315 | -0.2646 | No |
| 78 | TMEM97 | na |  |  | 12724 | -0.318 | -0.2631 | No |
| 79 | PTS | na |  |  | 12846 | -0.335 | -0.2669 | No |
| 80 | DCXR | na |  |  | 12972 | -0.336 | -0.2709 | No |
| 81 | CROT | na |  |  | 13059 | -0.351 | -0.2727 | No |
| 82 | ACO2 | na |  |  | 13111 | -0.355 | -0.2727 | No |
| 83 | PSMB10 | na |  |  | 13119 | -0.356 | -0.2704 | No |
| 84 | PC | na |  |  | 13181 | -0.366 | -0.2708 | No |
| 85 | UPB1 | na |  |  | 13272 | -0.382 | -0.2726 | No |
| 86 | DDT | na |  |  | 13433 | -0.408 | -0.2779 | No |
| 87 | NINJ1 | na |  |  | 13435 | -0.408 | -0.2749 | No |
| 88 | PDK4 | na |  |  | 13709 | -0.442 | -0.2859 | No |
| 89 | FAS | na |  |  | 13787 | -0.456 | -0.2865 | No |
| 90 | PTGR1 | na |  |  | 14155 | -0.500 | -0.3019 | No |
| 91 | GSTT2 | na |  |  | 14174 | -0.504 | -0.2990 | No |
| 92 | ECH1 | na |  |  | 14216 | -0.507 | -0.2973 | No |
| 93 | BPHL | na |  |  | 14231 | -0.510 | -0.2942 | No |
| 94 | PGRMC1 | na |  |  | 14268 | -0.516 | -0.2922 | No |
| 95 | SMOX | na |  |  | 14278 | -0.516 | -0.2887 | No |
| 96 | TMBIM6 | na |  |  | 14679 | -0.586 | -0.3053 | Yes |
| 97 | ARG2 | na |  |  | 14728 | -0.593 | -0.3033 | Yes |
| 98 | DHRS1 | na |  |  | 14794 | -0.602 | -0.3021 | Yes |
| 99 | GCH1 | na |  |  | 14797 | -0.603 | -0.2977 | Yes |
| 100 | VNN1 | na |  |  | 14949 | -0.628 | -0.3008 | Yes |
| 101 | ALDH3A1 | na |  |  | 14974 | -0.629 | -0.2973 | Yes |
| 102 | JUP | na |  |  | 15173 | -0.664 | -0.3027 | Yes |
| 103 | CDA | na |  |  | 15208 | -0.671 | -0.2994 | Yes |
| 104 | EPHX1 | na |  |  | 15301 | -0.687 | -0.2990 | Yes |
| 105 | ADH5 | na |  |  | 15308 | -0.688 | -0.2941 | Yes |
| 106 | ABHD6 | na |  |  | 15533 | -0.728 | -0.3003 | Yes |
| 107 | ACOX1 | na |  |  | 15588 | -0.740 | -0.2975 | Yes |
| 108 | ACOX2 | na |  |  | 15595 | -0.742 | -0.2922 | Yes |
| 109 | PDLIM5 | na |  |  | 15884 | -0.799 | -0.3012 | Yes |
| 110 | TNFRSF1A | na |  |  | 16016 | -0.823 | -0.3019 | Yes |
| 111 | IGFBP1 | na |  |  | 16061 | -0.836 | -0.2978 | Yes |
| 112 | ALAS1 | na |  |  | 16248 | -0.886 | -0.3009 | Yes |
| 113 | AKR1C3 | na |  |  | 16300 | -0.898 | -0.2967 | Yes |
| 114 | PTGES3 | na |  |  | 16323 | -0.904 | -0.2910 | Yes |
| 115 | CYB5A | na |  |  | 16333 | -0.907 | -0.2846 | Yes |
| 116 | ALDH9A1 | na |  |  | 16400 | -0.923 | -0.2811 | Yes |
| 117 | SPINT2 | na |  |  | 16414 | -0.926 | -0.2747 | Yes |
| 118 | ALDH2 | na |  |  | 16573 | -0.962 | -0.2757 | Yes |
| 119 | GSR | na |  |  | 16589 | -0.967 | -0.2691 | Yes |
| 120 | MT2A | na |  |  | 16590 | -0.967 | -0.2618 | Yes |
| 121 | TGFB2 | na |  |  | 16607 | -0.972 | -0.2553 | Yes |
| 122 | SLC46A3 | na |  |  | 16629 | -0.978 | -0.2489 | Yes |
| 123 | DDAH2 | na |  |  | 16863 | -1.045 | -0.2532 | Yes |
| 124 | CFB | na |  |  | 16882 | -1.049 | -0.2462 | Yes |
| 125 | ACP1 | na |  |  | 16940 | -1.069 | -0.2411 | Yes |
| 126 | ETFDH | na |  |  | 16947 | -1.071 | -0.2333 | Yes |
| 127 | MARCH6 | na |  |  | 16952 | -1.073 | -0.2253 | Yes |
| 128 | NPC1 | na |  |  | 17307 | -1.184 | -0.2349 | Yes |
| 129 | GSTM4 | na |  |  | 17368 | -1.204 | -0.2289 | Yes |
| 130 | SAR1B | na |  |  | 17410 | -1.220 | -0.2218 | Yes |
| 131 | ELOVL5 | na |  |  | 17447 | -1.235 | -0.2143 | Yes |
| 132 | BLVRB | na |  |  | 17508 | -1.262 | -0.2079 | Yes |
| 133 | MAOA | na |  |  | 17610 | -1.299 | -0.2033 | Yes |
| 134 | PTGES | na |  |  | 17682 | -1.324 | -0.1970 | Yes |
| 135 | MCCC2 | na |  |  | 17698 | -1.331 | -0.1877 | Yes |
| 136 | LPIN2 | na |  |  | 18001 | -1.486 | -0.1923 | Yes |
| 137 | ARPP19 | na |  |  | 18042 | -1.508 | -0.1829 | Yes |
| 138 | IL1R1 | na |  |  | 18099 | -1.548 | -0.1741 | Yes |
| 139 | PGD | na |  |  | 18151 | -1.582 | -0.1647 | Yes |
| 140 | CYP27A1 | na |  |  | 18279 | -1.661 | -0.1588 | Yes |
| 141 | GSTO1 | na |  |  | 18377 | -1.735 | -0.1507 | Yes |
| 142 | HPRT1 | na |  |  | 18443 | -1.794 | -0.1405 | Yes |
| 143 | ITIH4 | na |  |  | 18605 | -1.930 | -0.1343 | Yes |
| 144 | CAT | na |  |  | 18668 | -2.014 | -0.1223 | Yes |
| 145 | HSD11B1 | na |  |  | 18677 | -2.024 | -0.1073 | Yes |
| 146 | IDH1 | na |  |  | 18690 | -2.035 | -0.0925 | Yes |
| 147 | UGDH | na |  |  | 18907 | -2.368 | -0.0858 | Yes |
| 148 | CASP6 | na |  |  | 18952 | -2.470 | -0.0694 | Yes |
| 149 | GABARAPL1 | na |  |  | 19071 | -2.800 | -0.0543 | Yes |
| 150 | PROS1 | na |  |  | 19188 | -3.994 | -0.0301 | Yes |
| 151 | KYNU | na |  |  | 19189 | -4.024 | 0.0005 | Yes |
Table: GSEA details [plain text format]

  

Fig 2: HALLMARK\_XENOBIOTIC\_METABOLISM      
 Blue-Pink O' Gram in the Space of the Analyzed GeneSet

  

Fig 3: HALLMARK\_XENOBIOTIC\_METABOLISM: Random ES distribution      
 Gene set null distribution of ES for **HALLMARK\_XENOBIOTIC\_METABOLISM**
